# Interprofessional Collaboration and Involvement of Parents in the Management of Painful Procedures in Newborns

**DOI:** 10.3389/fped.2020.00394

**Published:** 2020-07-23

**Authors:** Colette Balice-Bourgois, Maya Zumstein-Shaha, Giacomo D. Simonetti, Christopher J. Newman

**Affiliations:** ^1^Pediatric Institute of Southern Switzerland, Ente Ospedaliero Cantonale, Bellinzona, Switzerland; ^2^Nursing Research Center, Ente Ospedaliero Cantonale, Bellinzona, Switzerland; ^3^University Institute of Higher Education and Research in Healthcare, Faculty of Biology and Medicine, University of Lausanne, Lausanne, Switzerland; ^4^Department of Health, Bern University of Applied Sciences, Bern, Switzerland; ^5^Faculty of Biomedical Sciences, University of Southern Switzerland, Lugano, Switzerland; ^6^Woman-Mother-Child Department, Lausanne University Hospital, Lausanne, Switzerland

**Keywords:** procedural pain management, neonatal pain, interprofessional collaboration, parental involvement, complex intervention

## Abstract

**Introduction:** Newborns are subject to many painful procedures. Pharmacological and non-pharmacological approaches alone are not enough, and it is necessary to consider other contributing elements such as the environment, interprofessional collaboration and parental involvement. The aim of this feasibility study was to explore interprofessionality and the role of parents in improving the management of painful procedures in newborns and pain management strategies.

**Materials and Methods:** a pre-post feasibility study using a mixed method approach was conducted. Questionnaires, interviews and focus groups were used to describe the parents' views on their child's pain management and involvement in care as well as to explore the level of interprofessionality and feasibility.

**Results:** Collaboration between physicians and nurses improved following the implementation of a complex interprofessional intervention involving professionals, parents and newborns. In spite of improving professional collaboration in procedural pain management, parents were attributed a passive role or only marginally involved in in the infant's pain management. However, parents stated—as elicited by the questionnaires and interviews—that they wished to receive more information and be included in painful procedures executed on their infant.

**Discussion:** Management of painful procedures in neonates needs to be changed. Interprofessional collaboration contributes to improved procedural pain management in neonates. It is essential to include parents as active members in the interprofessional healthcare team.

## Introduction

Newborns, especially premature newborns, are a patient group that is highly vulnerable to pain. Numerous studies have shown that newborns are subjected to many painful procedures in a stressful environment during their neonatal hospitalization (1–6). Despite important progress in the understanding of neonatal pain and the management of procedural pain, procedural pain is still poorly managed, and a gap remains between research and the translation of knowledge into practice. A systematic review of guidelines (7), has shown that the prevention of painful procedures includes not only pharmacological and non-pharmacological treatments but also parents and interprofessional collaboration. It is also essential to consider the environment (8).

In procedural pain management, many interfaces related to skills and tasks can be identified. Healthcare professionals' attitudes and beliefs, and parents' lack of knowledge and information can influence and limit optimal pain management. Pain relief constitutes an ethical foundation of the nursing profession, yet it is not always given adequate priority (9–11). Nurses have the most contact with patients. Their constant presence with newborns allows them to observe, assess, prevent and treat pain (12). Nurses, therefore, play a critical role in pain management, especially for the neonate and the premature infant, who are more vulnerable to pain and its short-term and long-term consequences because of their immature central nervous system (10, 13). Besides nurses, all members of the healthcare team play an important role in pain management as they have complementary roles and responsibilities that enhance patient care. In interprofessional care, all healthcare professionals should have a common goal and treatment decisions emerge from consensus (14). In neonatal care, interprofessional management not only includes health professionals, but also parents (15, 16). Teamwork has been shown to be a prerequisite for parental involvement in the management of infant pain (17). Medical hierarchies that limit autonomous decision making can negatively influence pain management practices, as well as the treatment preferences of individual physicians (18). Greater collaboration between nurses and doctors leads to improved care (19, 20). Parents can act as advocates for their child by advocating for the treatment of pain (21). Parental involvement has been shown to have short- and long-term benefits for both newborns and parents (22, 23). Parental presence can also improve neonatal pain care, for example by increasing the use of non-pharmacological methods of pain relief for painful procedures and the documentation of pain assessment (24, 25). In addition, parents express their willingness to be more involved in their infant's pain care (22, 26–29). It is therefore essential that parents take an active role in the management of their newborn's pain, but for this to happen it is necessary to increase information on pain and pain management as well as encouragement and inclusion from professionals (8, 22, 30). Therefore, an interprofessional approach that integrates the knowledge, skills and expertise of different professionals and the experience of parents could contribute to improved pain management in neonates. Communication and collaboration among professionals and parents, education as well as documentation of care constitute key elements of the interprofessional approach (14). The attitudes and beliefs of health professionals play an important role in the inclusion of parents during painful procedures (17, 31, 32). Much evidence exists on the inclusion of parents in painful care, but has yet to be fully implemented in clinical practice (24, 33). Therefore, interprofessional collaboration, which includes parents is essential for effective pain management practices (18, 19).

The aim of this feasibility study, was to explore interprofessionality and the role of parents in improving the management of painful procedures in newborns and pain management strategies.

## Materials and Methods

### Study Design

**Figure 1:**
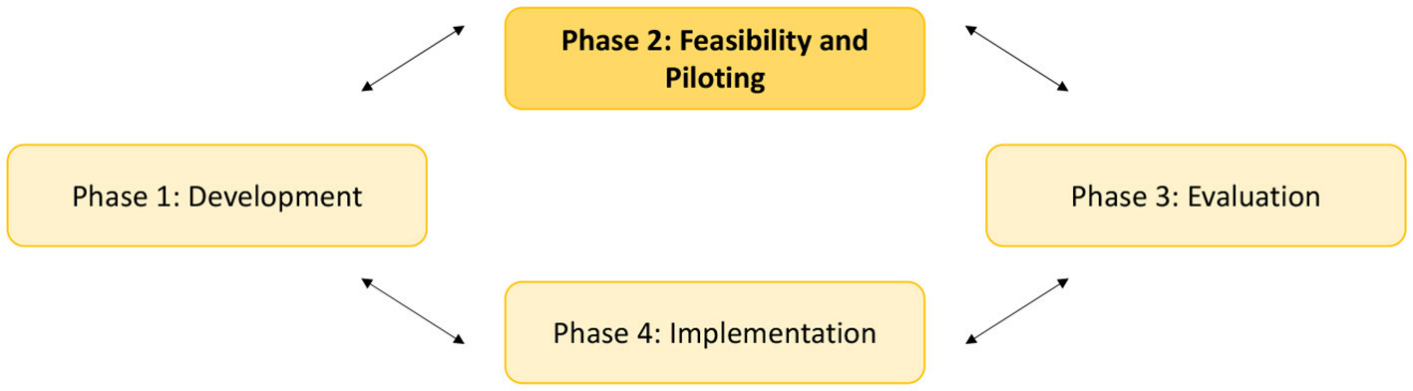
Key elements of the development and evaluation process: The Medical Research Council guidance (35).

A pre-post study with a mixed-method approach was used. A mixed method design was chosen because it has the advantage of increasing the understanding of how interventions should be structured, describing the context and promoting better interpretation of results (34–36). We followed phase two on the revised MRC guidelines (Figure 1) (35, 38) to test the feasibility of the NEODOL intervention (39). A set of results was produced (i.e., acceptability among parents and professionals, barriers, facilitators, adaptation for implementation), which help determine whether the intervention is feasible (40, 41). Questionnaires and focus groups were used to explore the level of interprofessionality among professionals. The parents' views on their child's pain management and their involvement in care was elicited via questionnaire and interviews.

### Setting and Sample

This feasibility study was conducted in a neonatal unit level IIb at a regional hospital in Southern Switzerland. This study was approved by the local ethics committee (CE TI 330). The neonatology unit includes a total of six beds with three intermediate care beds and three specialized care beds. According to unit statistics, 128 newborns were admitted in 2018 representing 878 hospital days.

### Description of the NEODOL Intervention

**Figure 2:**
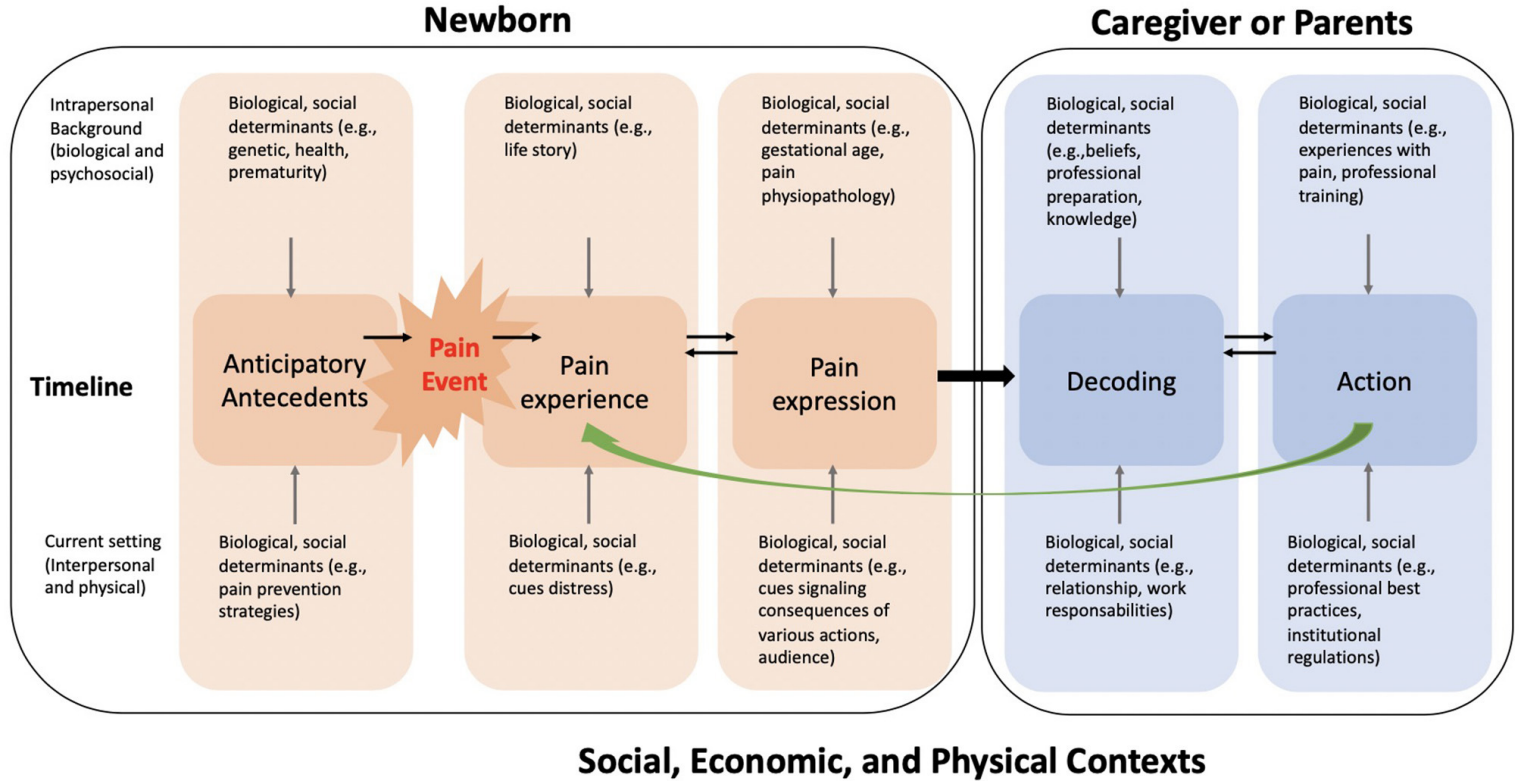
Social Communication model of pain - Craig KD [adapted from (37)].

In a previous study, we reported the development of the NEODOL intervention in accordance with phase one of the MRC guidelines (Medical Research Council) (39). The NEODOL intervention (39) is based on Craig's Social Communication Model of pain (37, 42) combined with recommendations drawn from a systematic review of guidelines (7). Craig's Social Communication Model of Pain (37, 42) (Figure 2) can provide insight into the complexity of pain management in newborns. According to this model, managing pain requires a multifaceted approach targeting healthcare professionals and parents. That's why this complex interprofessional intervention targets three groups: healthcare professionals (i.e., physicians and nurses), parents, and neonates of a neonatal unit. The intervention encompasses various components for each group (Table 1). For healthcare professionals, educational elements were combined with documentation on procedural pain to improve knowledge and interprofessional collaboration. Parents received written information on procedural pain management and to improve involvement during painful procedures. For newborns, a bundle of procedures was formulated to be applied by healthcare professionals prior to the painful procedure to reduce pain as much as possible (39). The bundle of procedures integrated six elements to be taken into account when performing a painful procedure: (1) Planning the procedure, (2) Collaboration and involvement of the family, (3) Environmental measures, (4) Pain assessment, (5) Choice of analgesia, and (6) Documentation in the patient's record.

**Table 1:** Study phases.

	**T0: Pre-intervention**	**T1: Intervention**	**T2: Post-intervention**	
	**Quantitative data**	**Intervention content**	**Quantitative data**	**Qualitative data**
	**collection**		**collection**	**collection**
HCP^*^	AICTS-II questionnaire	Interprofessional education Booklet & reminders Pain champion	AICTS-II questionnaire	Focus group
Parents	PAIN questionnaire	Parents' information leaflet	PAIN questionnaire	Parents' interview
Neonates				
		Implementation of the bundle of care for painful procedures		

* *HCP, health care professionals*.

### Study Overview

The study is divided into 3 phases: pre-intervention (T0), implementation of the NEODOL intervention (T1), and post-intervention (T2). The total duration of the T1 intervention was 5 months (Table 1).

### Implementation Strategy

The NEODOL intervention was implemented for a feasibility study. First, the healthcare professionals (i.e., nurses, physicians, pediatricians, and anesthetists) received a structured interprofessional education program (based on the “International Association for the Study of Pain-IASP Interprofessional Pain Curriculum Outline”) (43). A booklet was provided with protocols on pain management along with a pain treatment algorithm. A nurse was positioned as pain champion to support change and reminders were also utilized (e.g., putting up posters and stickers in the unit). Second, parents were given a leaflet informing them about procedural pain during hospitalization and how they could collaborate during these painful procedures. Third, a bundle of procedures to manage painful procedures in newborns was implemented containing all elements for each procedure. The bundle replaced the standard of care. Besides the pain champion, no additional staffing resources were required.

### Data Collection

#### Quantitative Section

The Italian version of the questionnaire “Assessment of Interprofessional Team Collaboration Scale” (AICTS-II) was used to assess the level of interprofessional collaboration (44–46). AICTS-II has a good internal consistency with a Cronbach alpha coefficient of 0.89 and for the Italian version it is between 0.92 and 0.94. The questionnaire consists of 23 items that are arranged into three subscales to measure partnership (8 items), cooperation (8 items), and coordination (7 items). Each item is rated using a 5-point Likert scale (from never = 1 to always = 5). The scores for the 3 domains were calculated by averaging the items in each domain, where values less than or equal to four indicate an inadequate perception of the domain (4.0 or more = good collaboration; 3.0 to 3.9 = moving toward collaboration; 1.0 to 2.9 = need to focus on developing collaborative practice). The questionnaire was submitted to healthcare professionals pre- and post-intervention.

The questionnaire “Parent Attitudes about Infant Nociception” (PAIN) (47) was used to describe parents' opinions related to their experience of managing their child's pain and their satisfaction. This questionnaire consists of 51 questions with a combination of yes/no, forced choice and Likert-type questions with additional space for comments. It has good internal consistency with a Cronbach's alpha of 0.71 for expectations and 0.84 for satisfaction. The questionnaire has been translated into Italian and validated according to Wild et al. (48). The questionnaire was submitted to parents pre- and post-intervention. However, parents were not the same in pre- and post-intervention.

For both questionnaires, permission for use was obtained from the authors (44, 46, 47).

#### Qualitative Section

Focus groups with healthcare professionals were conducted to determine intervention acceptability and feasibility (barriers and facilitators to the implementation) and to gather suggestions for refining the NEODOL intervention to render it more user-friendly. An interview-guide was developed addressing the experience with the NEODOL intervention and difficulties encountered. Participants were invited to provide feedback on interprofessional collaboration between physicians and nurses as well as on the parents' involvement. These focus groups were conducted at the end of the 5-month pilot period.

Semi-directed interviews with parents were conducted and recorded. The parents had a child that was hospitalized in the neonatology unit. In order to facilitate the organization of the interviews, they were conducted only with the mothers and in their homes after the infant was discharged. An interview guide was developed that addressed the experience of painful procedures, concerns about pain and parental involvement in care.

In order to partially minimize social desirability bias, the focus groups and interviews were conducted by persons who were not involved in the research but were trained in qualitative methods.

### Data Analysis

#### Quantitative Section

Data analysis were performed using SPSS software (IBM, Version 25). For each of the questionnaires, due to the small sample, only descriptive analyses were performed.

For AICTS-II, scores for the different domains (partnership, coordination, cooperation) were calculated by the mean of the items in each domain, where values less than or equal to four indicate an inadequate perception of the domain of interprofessional collaboration (44, 45). Mann–Whitney *U*-tests were used for evaluating differences in AICTS scores between nurses and physicians.

#### Qualitative Section

Data analysis were managed using MAXQDA software (version 18). The interviews and focus group were transcribed, and thematic analysis was performed (49). This analytical process involves reading and re-reading the transcripts, identifying units of meaning using codes, and then organizing the data into broader themes representing the main ideas. The data were coded by the principal investigator and a research collaborator. The codes were then discussed and compared. A final consensus was reached by the inclusion of a third researcher. A final list of codes was created based on the consensus. Any discrepancies in coding were discussed and resolved between the three researchers.

## Results

### AICTS-II Questionnaire

The questionnaire was distributed to a total of 60 professionals in T0 and T2. The response rate in T0 was 78.3% (*N* = 47) and in T2 was 60% (*N* = 37) (Table 2). Average age of the professionals was 36 years ± 8.8 years for the T0 group and 35 years ± 8.7 years for the T2 group. On average, professionals had worked 11.5 years in T0 and 10.69 years in T2, and in the current care unit 6.68 years in T0 and 5.64 years in T2.

**Table 2:** Demographics of healthcare professionals (HCP) for the AICTS-II.

	**Pre-intervention (T0)**		**Post-intervention (T2)**	
**Variable**	***n***	**%**	***n***	**%**
Response rate	47	78.3	36	60
Gender				
Male	14	29.8	8	22.2
Female	33	70.2	28	77.8
Profession				
Nurse	27	57.4	18	50
Physician	20	42.6	18	50

The use of AICTS-II questionnaire provided baseline information on staff attitudes and perceptions of interprofessional collaboration. Staff perceptions of team functioning before and after the NEODOL intervention was also determined. The results of the AICTS total and subscales among all participants as well as in subgroups are presented in Table 3. Results show that nurses have a level below 4 and physicians have a level above 4 except for the pre-intervention “coordination” domain. Statistically significant differences between the two groups (nurses and physicians) were found pre and post intervention for the AICTS Partnership and Cooperation subscales and for the total AICTS score. For the coordination subscale, no statistically significant differences were found pre and post intervention for each group (nurses and physicians). However, an increase in each subscale was observed in the physician group as well as for interprofessional collaboration. Similar results were observed for the nurses pre and post intervention.

**Table 3:** Subscales scores and total scores for the AICTS-II.

	**Pre-intervention (T0)**				**Post-intervention (T2)**			
	Total HCP^**^	**Nurses**	**Physicians**		Total HCP^**^	**Nurses**	**Physicians**	
	**(*N* = 47)**	**(*N* = 27)**	**(*N* = 20)**	P^*^	**(*N* = 36)**	**(*N* = 18)**	**(*N* = 18)**	P^*^
	**Mean (*SD*)**	**Mean (*SD*)**	**Mean (*SD*)**		**Mean (*SD*)**	**Mean (*SD*)**	**Mean (*SD*)**	
Partnership	3.85 (0.65)	3.66 (0.68)	4.10 (0.52)	**0.029**	4.05 (0.76)	3.70 (0.78)	4.39 (0.57)	**0.008**
Cooperation	3.96 (0.50)	3.86 (0.36)	4.10 (0.62)	**0.043**	3.99 (0.81)	3.67 (0.75)	4.30 (0.76)	**0.029**
Coordination	3.75 (0.54)	3.63 (0.39)	3.91 (0.68)	0.059	3.86 (0.84)	3.67 (0.69)	4.06 (0.95)	0.085
AICTS-total (Interprofessional collaboration)	3.85 (0.48)	3.72 (0.38)	4.04 (0.55)	**0.013**	3.96 (0.77)	3.68 (0.71)	4.25 (0.73)	**0.024**

*Bold values indicate statistically significant differences*.

* *Mann–Whitney U-test were used for evaluation differences in AICTS between nurses and physicians*.

** *HCP, healthcare professional*.

### Focus Groups With Healthcare Professional

A total of three focus groups were conducted. In two focus groups doctors and nurses both participated. One focus group consisted of nurses only with different levels of professional experience. In total, 20 healthcare professionals (13 nurses and 7 physicians) participated in the focus groups. The mean age was 36.2 years (range 28–56).

A total of six categories emerged: “*Increasing awareness of pain*,” “*Interprofessional collaboration*,” “*Parental involvement*,” “*Culture change*,” “*Facilitators to using NEODOL*” and “*Barriers to using NEODOL*.” Physicians and nurses generally reported positive outcomes from implementing the NEODOL intervention.

#### Increasing Awareness of Pain

Interprofessional education was highly regarded and considered to increased knowledge among both nurses and physicians. A more reflective and collaborative practice has developed as a result of this awareness, as one nurse commented that the NEODOL intervention has given more attention than before to perform certain types of care. A physician noted: “NEODOL (.) has helped many people to understand that there are various ways of working. There is one way, as I have often heard, out of habit, and there is, in contrast, a wise way of working. So, when I enter an environment where you work out of habit, I learn to work out of habit, and I do not take into account other factors. NEODOL raises awareness to consider: “every time I prescribe something, every time I take action, I have to rationalize why I do it, how I do it, and when I do it'.”

#### Interprofessional Collaboration

Nurses and physicians reported positive experiences of interprofessional collaboration with better communication and collaboration in organizing pain care. One nurse stated: “A positive experience was also working in collaboration with physicians. Maybe, you had a blood test in the morning, (.) at 7.30 am. And you knew your newborn that all care was planned for 9 am. And if it was not an urgent blood test, of course, I always encountered positive collaboration.”

#### Culture Change

In each focus groups, the participants spoke about their work in a way that revealed their commitment to managing procedural pain better. This was a common thread throughout. In one of the focus groups of nurses, the importance was described as follows: “I feel like I am doing many things that we used to do before anyway, but without even realizing it. The fact is, they are now documented.” Another nurse commented: “With NEODOL, care is more patient-centered (.) It is no longer based on the organization of the ward (.) There is clinical reasoning (.) And you realize it is feasible. It is not impossible.

#### Parental Involvement

Differences in professional attitudes toward parental involvement in pain management were observed. A few nurses practiced parental involvement and found it to be a positive experience. For others, parental involvement was difficult because the professionals were afraid of parental judgment, of not succeeding or simply out of routine/habit. “So my perception is that sometimes (.) the parents are not involved (.), because you are a little afraid of the judgment, or the pressure that a parent can eh rightly have against you because you are doing a painful procedure on the child, to their child (…) In my opinion, when there is too little experience, there is a tendency perhaps not to call the parent, to do the procedure on your own, without feeling the additional pressure from the parents.” Other nurses reported that parents were too stressed, and that it was their duty as nurses to protect the parents from their child's pain. Parental involvement was controlled by the nurses and not in partnership with the parents. A nurse said: “If the parent is already anxious and we say: ‘hold your child in your arms and we will do the bloodwork from the heel', the parents start to get more anxious. Then, the babies become agitated as they feel the agitation and anxiety of their mom or dad. And in that case, maybe it is better that the parents were not involved (laughter). Honestly, it would be better in situations like these that I give the baby a pacifier with glucose and swaddle him/her in the blanket, so the baby stays calm and quiet. In times like these, it is more difficult and time-consuming to involve the parents.” One physician reported that parental involvement depended on the type of procedure performed.

#### Facilitators to Using the NEODOL

The reminders were considered to be facilitators for the implementation. The pain champion promoted change. This role was considered invaluable for promoting the use of the NEODOL intervention in practice. The pain champion was locally based, accessible and also understood the contextual complexities of the unit such as the workload, culture and challenges specific to the patient population.

#### Barriers to Using the NEODOL

Organizational resources constituted the main barriers, namely time, resources and workload. “There are situations, in which things have gone very well but (.) it depends a lot on both the parents and the time you have (.) especially (.) the number of patients you have, in order to devote time to the babies. It is true that there have been situations where painful procedures have been done and the child has not felt anything. This (.) in my opinion really needs time to do everything.”

### PAIN Questionnaire

Before the intervention was implemented, 10 parents completed the PAIN questionnaire. A total of 14 other parents responded to the questionnaire after the implementation. Parents rated their infant's most severe pain on a scale of 1 to 10–no pain to severe pain—with an average of 4.4 (SD 2.55) before the introduction of NEODOL and 3.93 (SD 2.81) after. Parents expected infants to experience less pain with 2.8 (SD 2.35) before implementation and 3.31 (SD 1.75) after. However, they also expected that a high degree of pain relief was provided to their infant. Parents, therefore, understood pain better after the NEODOL intervention.

Before the NEODOL intervention, parents (*n* = 7/10; 70%) reported that healthcare professionals had never or not often asked them about their preferences to be present or not during painful procedures. After the intervention, (*n* = 7/14; 50%) of the parents had not been asked about their preferences. The presence of parents during painful procedures seemed higher post-intervention. In pre-intervention, only 30% (*n* = 3/10) of parents reported that they were shown how to identify their infant's signs of pain but 64% (*n* = 9/14) in post-intervention. A total of 18 parents out of 24 (75%) had responded to the PAIN questionnaire (pre and post intervention). Most of these comments (*n* = 17/18, 94%) indicated that they wanted to be involved “always” or “as much as possible” and receive more information about their baby's pain.

### Interviews With Parents

A total of four interviews were conducted with an average duration of 37 min. Mothers' mean age was 35 years (range 28–41), that of the fathers was 39 years (range 28–51). The newborns were two girls and two boys with a mean gestational age of 36.25 (range 30–40). Their mean birth weight was 2,531 g (range 1400–3175).

Analysis yielded five main themes. The first concerns the experience of being a parent in neonatology. The second is the appreciation of professional skills. The third relates to infant procedural pain as a source of parental stress. The fourth describes the parental involvement in painful procedures and the last one concerns the NEODOL intervention.

#### The Experience of Being a Parent in Neonatology

Parenting in neonatology is a major challenge because there is much uncertainty. Parents are destabilized by this difficult experience and experience guilt. One mother explains: “The hardest moment, (.) is when they took me out and I had to leave the baby. I had a knot in my throat. That was the only time I started crying and (.) I had a hard time (bitter laughter). Because (…) it is very hard to have to go home and leave the baby there.” The neonatal unit was described as having limited space and privacy. A strict schedule for care existed, which did not make the parents' experience any easier. Another mother reported: “It was clearly not (.) easy for us parents, because it is the first child. The first experience so (.)we did not know how to behave.”

#### Infant Procedural Pain as a Source of Parental Stress

The mothers described their distress at the sight of various procedures or technical acts such as insertion of nasogastric tubes or heel punctures. Observing their baby being treated was difficult: “The first time I saw (.) the catheter. Putting the tube in is the hardest part (.) It really bothered the baby. I interpreted it as painful for the baby.”

#### The Appreciation of Professional Skills

Parents had confidence in the clinical staff and were satisfied with the care. The parents were grateful for the professionalism of the nurses and doctors. Professional support and assistance were reassuring to parents. One mother stated: “I must say that they always took their time, for example they warmed her foot, they really tried to be as minimally invasive as possible; they waited until she was calm. They did everything calmly, with a lot of patience. I appreciate that.”

#### Parental Involvement in Painful Procedures

Parents wanted to be involved in their child's care. However, they needed adequate information in order to support their child during a painful procedure. Generally, parents had a passive role such as simply being present at their child's side: One mother recounted: “And then he was crying a lot, so I stood there just to make him feel the warmth. (.) They gave him shots (…) almost every morning (.)he was in pain, suffering (.) that is what I thought.” Another mother pointed out that she was not present during various painful procedures: “Then, they put it [the nasogastric tube] in his nose. They did not call me in, because I am sure he will have cried (bitter laughter). I only wondered about that afterwards when [the nurse] told me, eh, he [baby] was pricked to have a venous line, (.) I said to myself, no, poor thing! They did not let me be of assistance. That is to say they would not let me come in because she would be crying very loudly. Poor thing, only just born (sighs). Maybe in these moments, they do not wish for parents to be present, and so they did not tell me. (…) I was not present, I did not see her cry.” Parents did not know what they could do to help or what their child needed. The parents wished for better information. Healthcare professionals should provide information and education on painful procedures, preferably in a calm environment. Such information should be offered without parents having to ask for it.

#### The NEODOL Intervention

Parents appreciated this intervention as it increased the sensitivity of the healthcare professionals. Through NEODOL, parents saw a chance to become more involved in their child's painful procedures. “The thing, at least from my point of view, … to know what they [healthcare professionals] are doing, to know how they [children] may feel while undergoing these treatments, to be involved as much as possible, to feel that I as a parent can help.” To increase parental involvement, it was suggested: “Just this: [as parents] to be better informed about the procedures. That it is it: to know when they do it, and at what time to be present.”

## Discussion

The purpose of this study was to explore interprofessionality in a pediatric neonatology ward, and the role of parents in improving the management of painful procedures in newborns. For the development of the NEODOL intervention, Craig's Social Communication Model of Pain proved to be an invaluable guide to understand the interactions between various dimensions. This model provided insight into the influence of biological, psychological and social factors as well as of the context on pain experience. Drawing on this model, these elements were included in NEODOL, thereby providing a multifaceted approach to improve procedural pain management in neonates.

In reporting this feasibility study, we followed the phase two of revised MRC guidelines (35, 38). Thus, we were able to gather information on acceptability and practicability of the NEODOL intervention (40, 50). Important lessons were learned from this study. Therefore, we reduced the risk of impracticality or methodological uncertainty and we helped refine intervention delivery characteristics (41).

A competent, collaborative, patient-centered, interprofessional team is needed to ensure the pain management related to procedures (51). However, the results of the AICTS questionnaire show that interprofessional collaborative practice still needs improvement. The lack of significant results may be due to an important decrease in participants in the post-intervention period. Work overload, lack of time, lack of clarity of the importance of participating in the research could explain the nurses' lower level of completion of the post-intervention questionnaire. For greater success, clarification of the importance of participation and more leadership could promote completion of the questionnaire. Interprofessional education for nurses and physicians increased knowledge and awareness on procedural pain management in healthcare professionals. Better care planning was obtained through better communication and collaboration between doctors and nurses. Nurse-physician collaboration constitutes an essential predictor to the provision of evidence-based pain management (19). In this study, healthcare professionals attended education on the NEODOL intervention, which included strategies to prevent procedural pain in neonates. Interprofessional collaboration is enhanced through strategies such as education for all healthcare professionals together or clarification of work processes (51, 52). Providing education on the same content to all healthcare professionals at the same time puts the knowledge level on an equal footing. Thus, healthcare professionals share the same educational experience (53, 54). Through NEODOL, all healthcare professionals had the same goal regarding procedures, namely, to improve pain management. Therefore, healthcare professionals not only had the same education, but also shared the same goal. Interprofessional education as well as having a common goal are key components to facilitate reflective and collaborative practice among professionals (14, 51, 55).

With the bundle of care, healthcare professionals reported being able to communicate and collaborate more effectively as a team (56). The presence of a healthcare professional acting as a champion for improving pain management constituted another important element to strengthen practices and collaboration among professionals (57). The team perceived this champion to be a major facilitator in the successful implementation of the NEODOL intervention.

NEODOL introduced parents as members of the interprofessional team as advocated by this recent systematic review (7). Parents were informed about procedural pain in the care of neonates. Similarly, healthcare professionals were educated about integrating parents into care. Interprofessional collaboration not only concerns healthcare professionals, but also needs to extend to parents and/or the patients themselves. Thus, interprofessional collaboration is strengthened (17, 21, 58, 59). However, various attitudes and experiences existed within the healthcare team on this study. Reasons were manifold and ranged from protecting parents from their child's pain to consideration of the type of procedure, difficulties planning procedures or the stress of nurses to performing painful procedures in the presence of parents. Some healthcare professionals found integrating the parents to be stimulating and were happy with the resulting collaboration. Nevertheless, healthcare professionals mostly maintained control on the decision to integrate parents as members of the interprofessional team. Axelin and al. described three different ways of working with parents for managing infant pain (31), which are: Nurses in control, Nurses sharing some control with parents and Nurse-parent collaboration. In the present study, the parents often remained in a passive role in pain management and, therefore, nurses were still very much in control. Further exploration is needed to determine the barriers and facilitators to integrating parents in procedural pain management.

Although parents are not present 24 h a day, they want to be better informed about their baby's care and to be involved in the management of neonatal pain as much as possible (27, 29, 60). The findings of this study corroborate this trend. In the interviews, parents expressed the difficulty and stress of being a parent in the NICU. Observing painful procedures was challenging as parents often did not know what do to or having an active role in pain management. However, parents were happy to observe the professionals' skills, which helped breach this gap. Few professionals asked parents for their preferences regarding presence during painful procedures at night. Parents expressed the wish to be more informed and integrated in the management of such procedures without having to ask for it expressively. An intervention addressing such needs, i.e., NEODOL, was perceived to be very important to improve their involvement. The findings from this study correspond with previous studies, which indicate that healthcare professionals need to recognize the parents' wish to be present during painful procedures. It is equally important to provide the information parents need to support their infants (22, 26, 61).

Craig's Social Communication Model of Pain, which has informed this study, highlights that pain must be approached in a multi-dimensional manner. All relevant stakeholders need to be included. The model provides a basis for conceptualizing interprofessional collaboration, which not only refers to healthcare professionals but also includes parents. For optimal management of painful procedures, collaboration between healthcare team members and parents is essential.

Several limitations of this study can be identified. Only one neonatology unit was involved in this feasibility and acceptability study. The participating pediatric unit consists of 19 beds, including six for neonatology. Recruitment was challenging. As the objective of this study was to determine feasibility and acceptability of the intervention (40, 50), small sample sizes are considered acceptable (62). The questionnaires used in this study had high validity and reliability in their original language version. The AICTS in Italian version shows good evidence of its validity and reliability (46). However, social desirability may have influenced completion of the AICTS as it is a self-report questionnaire. The essential steps for the development of a high quality and linguistically valid translation following Wild (48) were carried out on the PAIN questionnaire (47). However, validity and reliability were not determined in this study of the Italian version. Given the need to understand perceptions and attitudes about interprofessional collaboration, the perspective of the healthcare professionals yielded valuable information about perceived strengths and areas of intervention. The opportunity in the focus group to have two mixed groups of professionals and one nurse-only group produced data with different content, reflecting the diverse concerns and the complementary nature of the professional roles (63). Through a stepwise, mixed-method procedure, we showed that the NEODOL intervention was acceptable for parents and health professionals to manage procedural pain of neonates in clinical practice. Considering all findings of this feasibility and acceptability study, NEODOL offers a way to change the culture to improve interprofessional collaboration and to include parents as members of the interprofessional team. However, a couple of modifications need to be introduced in the modeling of the final intervention to facilitate implementation, namely editing the information to parents and to simplify electronic documentation. Further research should include obtaining valid and accurate estimates of the effect of the NEODOL intervention in clinical practice, which constitutes the next phase of the revised MRC guidelines (35, 38).

## Conclusion

We found that the NEODOL intervention targeting all groups (i.e., health professionals, parents and newborns) constitutes an innovative way to demonstrate the importance of interprofessional collaboration in the management of painful procedures. This feasibility and acceptability study shows that we are moving in the right direction and highlights the need for further research.

## Data Availability Statement

The datasets presented in this article are not readily available because of patient confidentiality and participant privacy. Requests to access the datasets should be directed to corresponding author Colette Balice-Bourgois.

## Ethics Statement

The studies involving human participants were reviewed and approved by Ethics Committee Tessin, Bellinzona (Swissethics). The patients/participants provided their written informed consent to participate in this study.

## Author Contributions

CB-B is the main author of the manuscript, organizing all aspects of the article including data extraction, drafting of the initial manuscript, and making revisions. MZ-S guided the conceptualization of the article and supervised drafting and revising of the manuscript. MZ-S, GS, and CN all provided guidance critically reviewed the manuscript. All authors contributed to manuscript revision, read and approved the submitted version.

## Conflict of Interest

The authors declare that the research was conducted in the absence of any commercial or financial relationships that could be construed as a potential conflict of interest.
